# Supplementary material for: Physiological ER stress caused by amylase production induces regulated Ire1-dependent mRNA decay in Aspergillus oryzae
Source: Commun Biol. 2023 Oct 4;6:1009. doi: 10.1038/s42003-023-05386-w (PMC10551036; doi:10.1038/s42003-023-05386-w)
Supplement: Supplementary file 2 — Supplementary Information [file 42003_2023_5386_MOESM2_ESM.pdf]

Supplementary information

Physiological ER stress caused by amylase production induces regulated Ire1-dependent mRNA decay in *Aspergillus oryzae*

Mizuki Tanaka[1,*], Silai Zhang[2], Shun Sato[2], Jun-ichi Yokota[2], Yuko Sugiyama[2], Yasuaki Kawarasaki[3], Youhei Yamagata[1], Katsuya Gomi[2,4,*], Takahiro Shintani[2,*]

Corresponding authors:

Mizuki Tanaka, mizuki-tanaka@go.tuat.ac.jp

Katsuya Gomi, katsuya.gomi.a6@tohoku.ac.jp

Takahiro Shintani, takahiro.shintani.d7@tohoku.ac.jp

This PDF file includes:

Supplementary Figures 1 to 10

Supplementary Tables 1 to 3

Supplementary Method

Supplementary Reference

Supplemental Figures

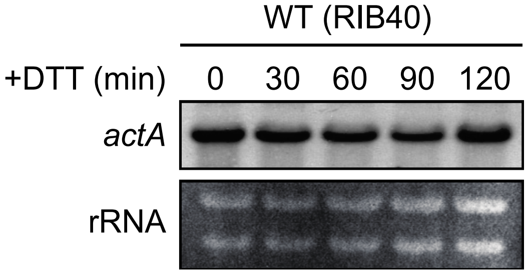

Supplementary Fig. 1. Northern blot analysis of the *actA* mRNA under ER stress condition. WT (RIB40) strain was grown in YPM medium for 24 h, following which DTT was added to the culture to a final concentration of 20 mM. After incubation for the indicated periods of time, mycelia were harvested and used for preparation of total RNA, which was subjected to northern blot analysis. Digoxigenin (DIG)-labeled DNA fragment of *actA* was synthesized as described in Supplementary method and was used as a probe.

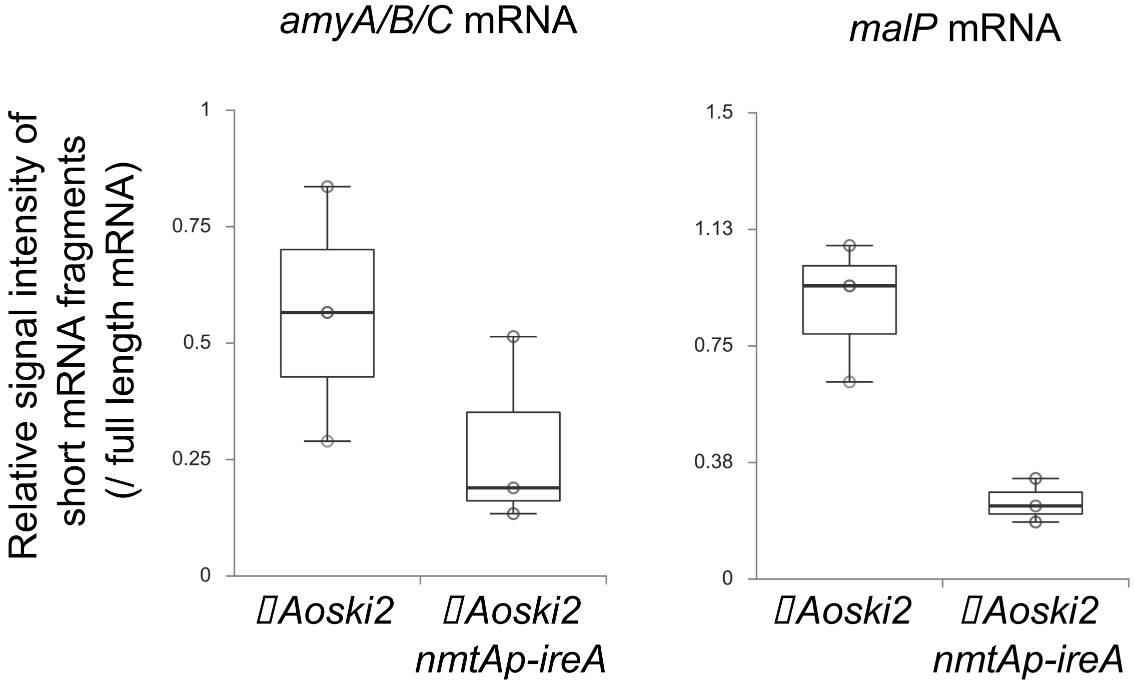

Supplementary Fig. 2. The relative amounts of short mRNA fragments to full-length mRNA. Signal intensities of *amyA/B/C* and *malP* mRNAs at 60 minutes following maltose addition were quantified using Image J software. The relative amounts of short mRNA fragments to full-length mRNA from three independent experiments are represented by boxplots. Box-plot elements: center line, median; box limits, upper and lower quartiles; whiskers, maximum and minimum values.

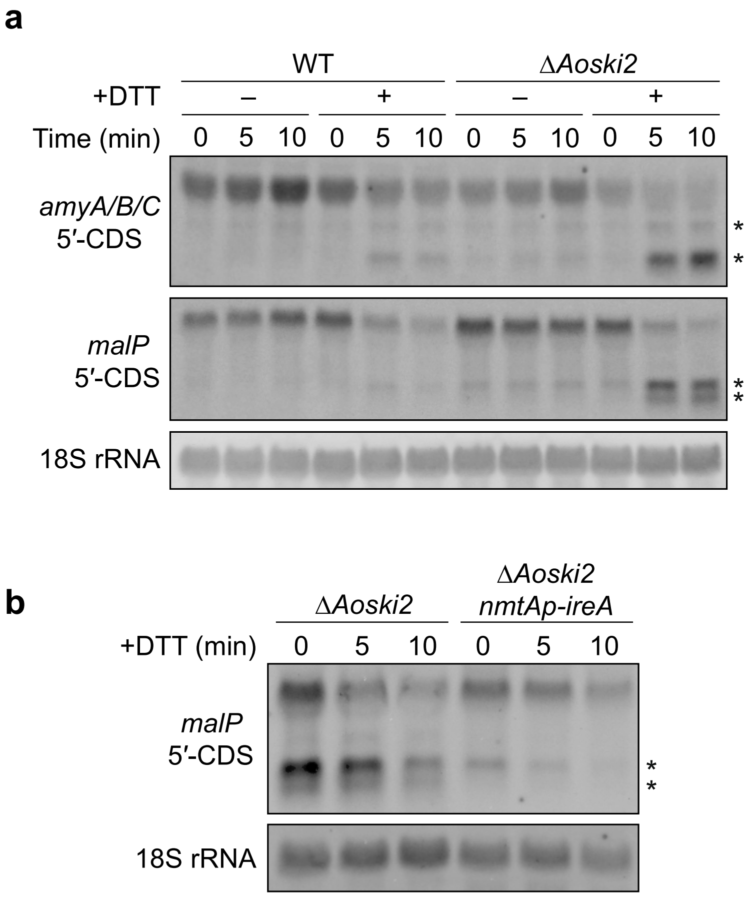

Supplementary Fig. 3. Involvement of ER stress in the cleavage of the *malP* mRNA. (a) Northern blot analysis of the *malP* mRNA in WT and Δ*Aoski2* strains treated with DTT or DMSO (solvent used for dissolving DTT). Both strains were pre-cultured in CD (glycerol) medium containing 0.1% polypeptone at 30°C for 24 h, and the mycelia were resuspended in CD (maltose) medium. After 60 min incubation at 30°C, DTT (20 mM final concentration) or an equal volume of DMSO was added to the medium, and mycelia were harvested at the indicated time for northern blot analysis. The asterisks indicate short mRNA fragments. (b) Northern blot analysis of the *malP* mRNA in the Δ*Aoski2* and Δ*Aoski2*/*nmtAp-ireA* strains treated with DTT. Both strains were grown and treated with DTT as shown in Fig. 3A for northern blot analysis. The asterisks indicate short mRNA fragments.

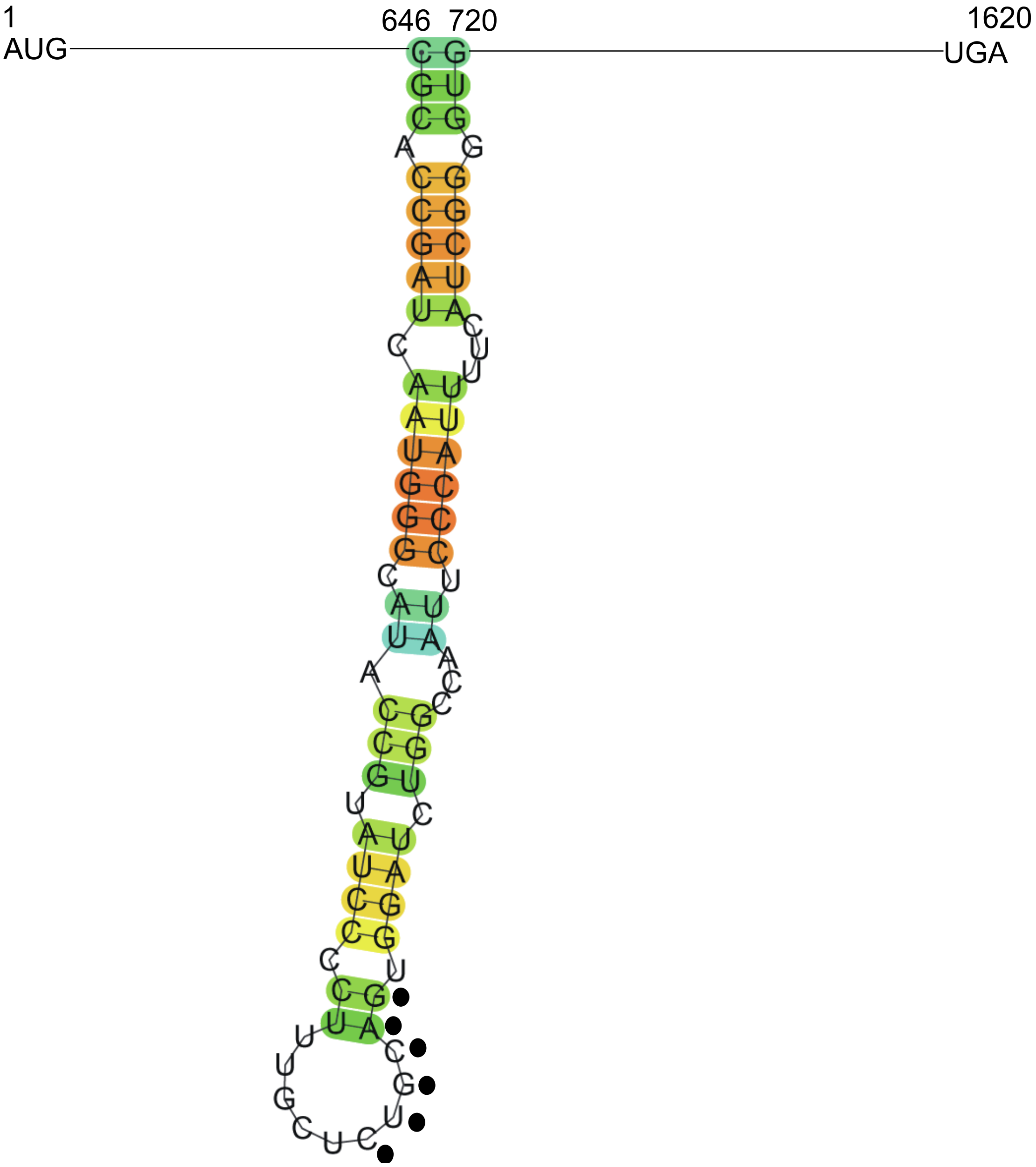

Supplementary Fig. 4. Predicted stem-loop structure within the *malP* mRNA coding region. The predicted secondary structure between nucleotides 646 to 720 obtained using CentroidFOLD (http://rtools.cbrc.jp/centroidfold/). The CUGCAG sequence is indicated by the black circles.

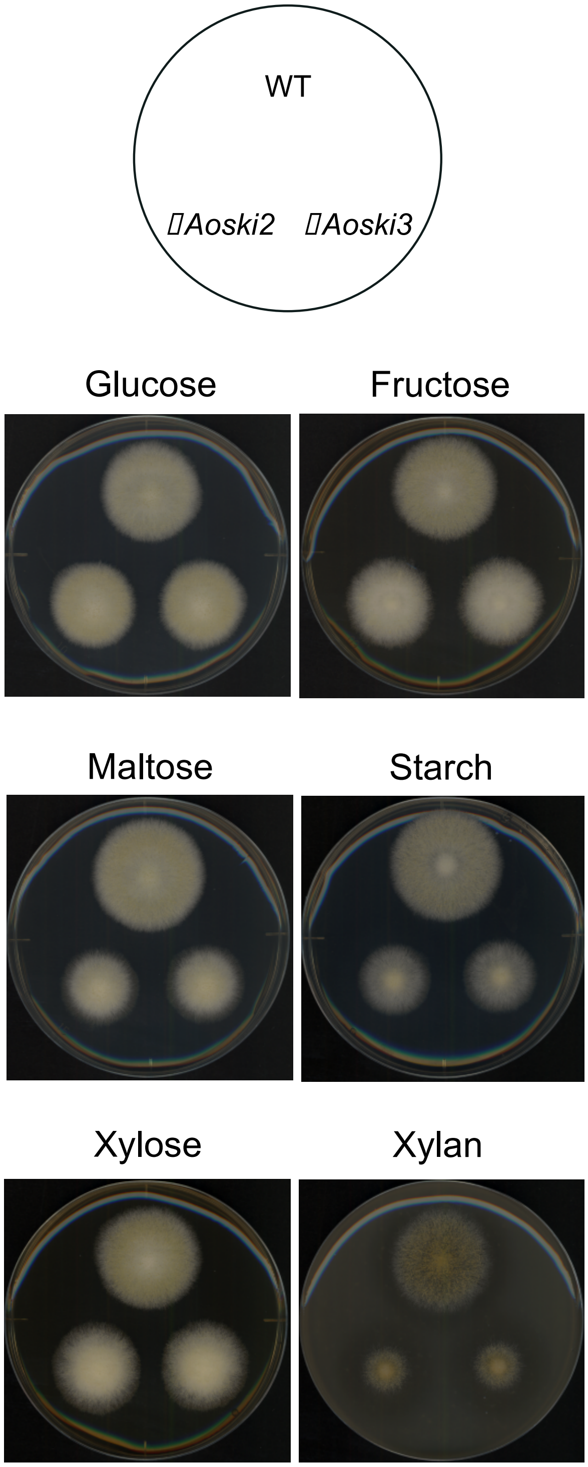

Supplementary Fig. 5. Growth of Ski complex-deficient strains. Approximately 1 × 10^4 conidiospores of each strain were grown for 4 days at 30°C on minimal agar media containing 1% indicated sugars as the sole carbon source.

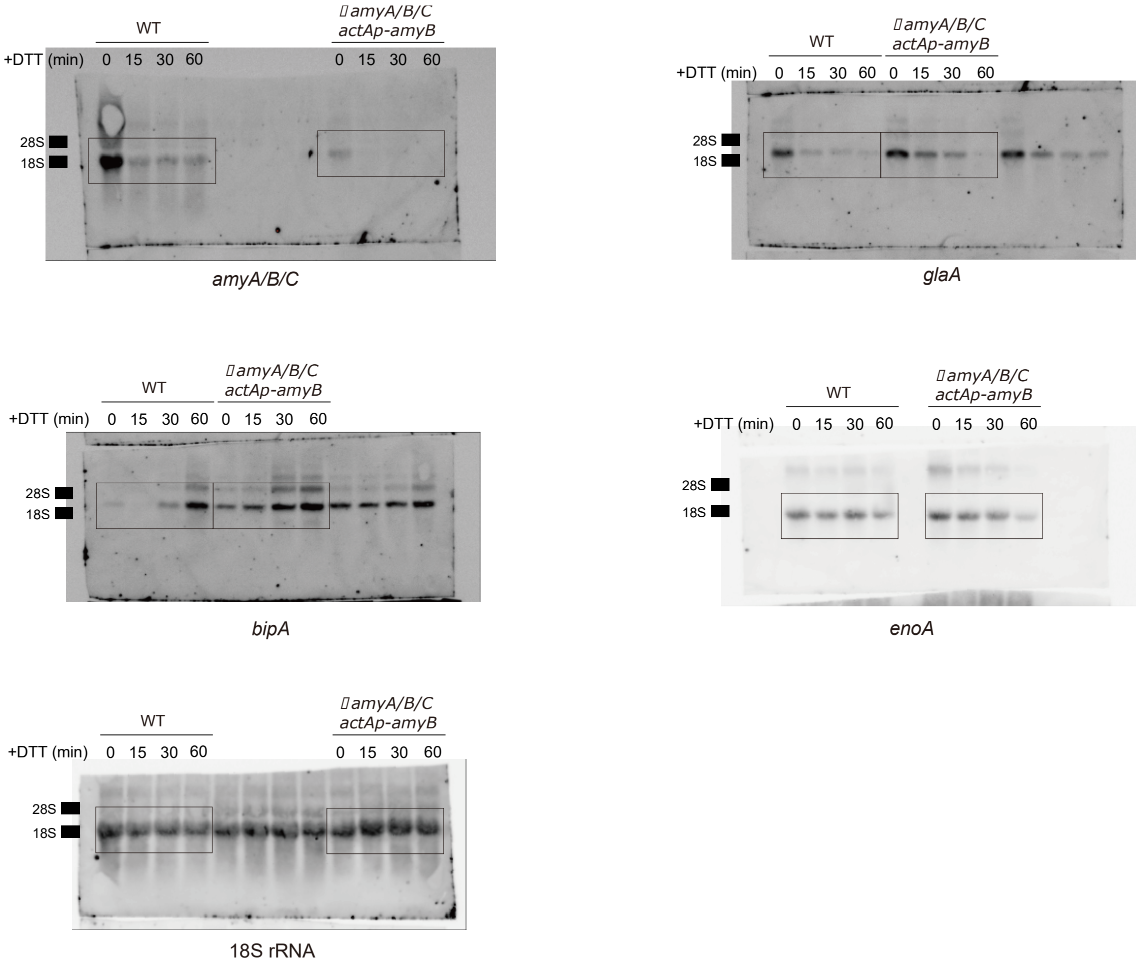

Supplementary Fig. 6. Uncropped and unedited northern blot images for Fig. 1. The positions of 28S rRNA (3,364 nucleotides) and 18S rRNA (1,799 nucleotides) stained by ethidium bromide are shown.

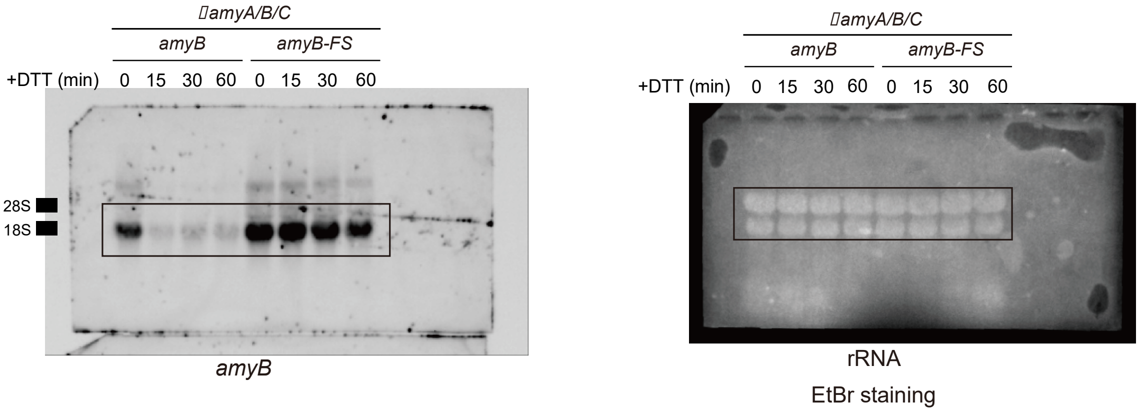

Supplementary Fig. 7. Uncropped and unedited northern blot images for Fig. 2c.

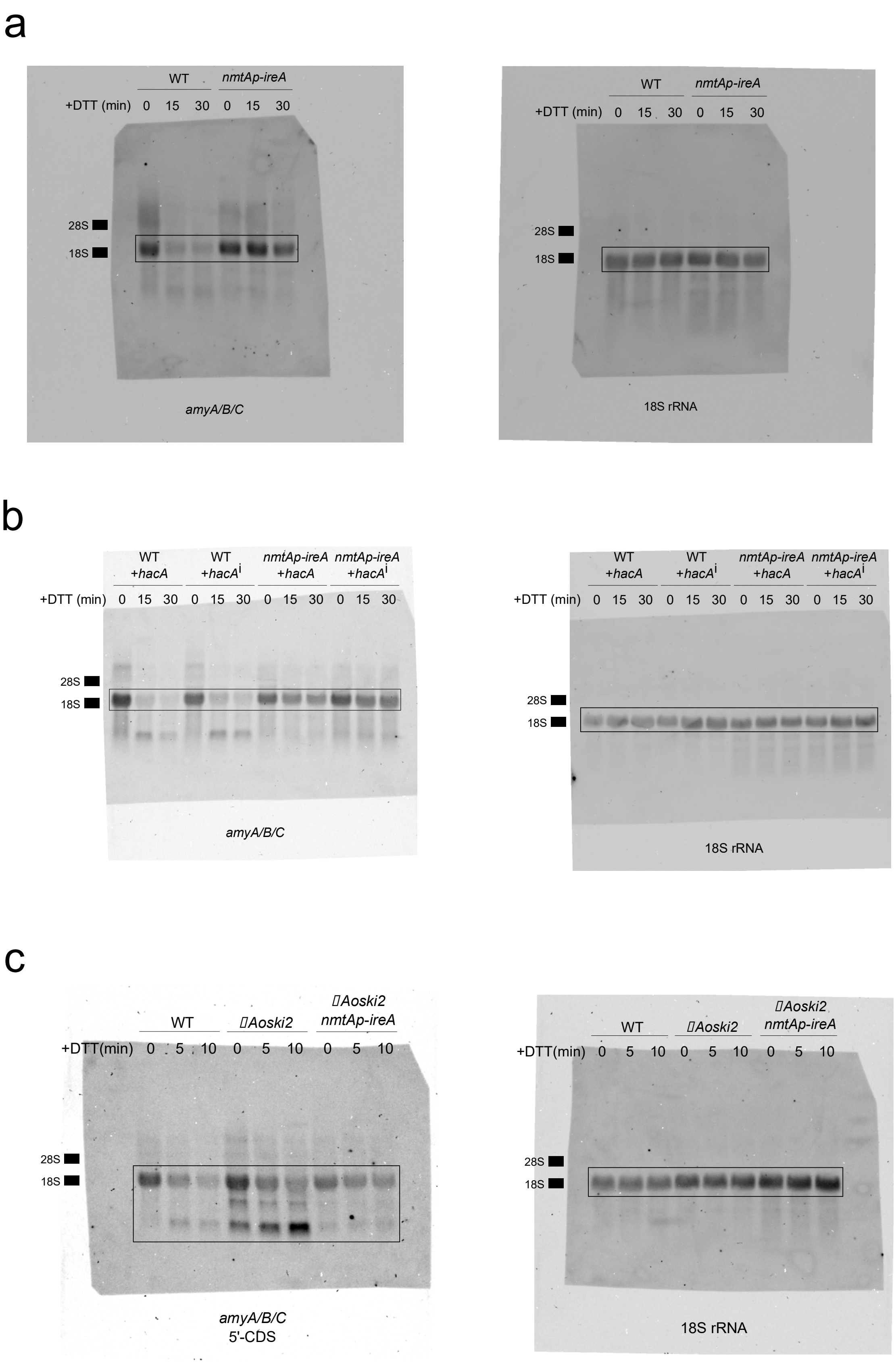

Supplementary Fig. 8. Uncropped and unedited northern blot images for Fig. 3.

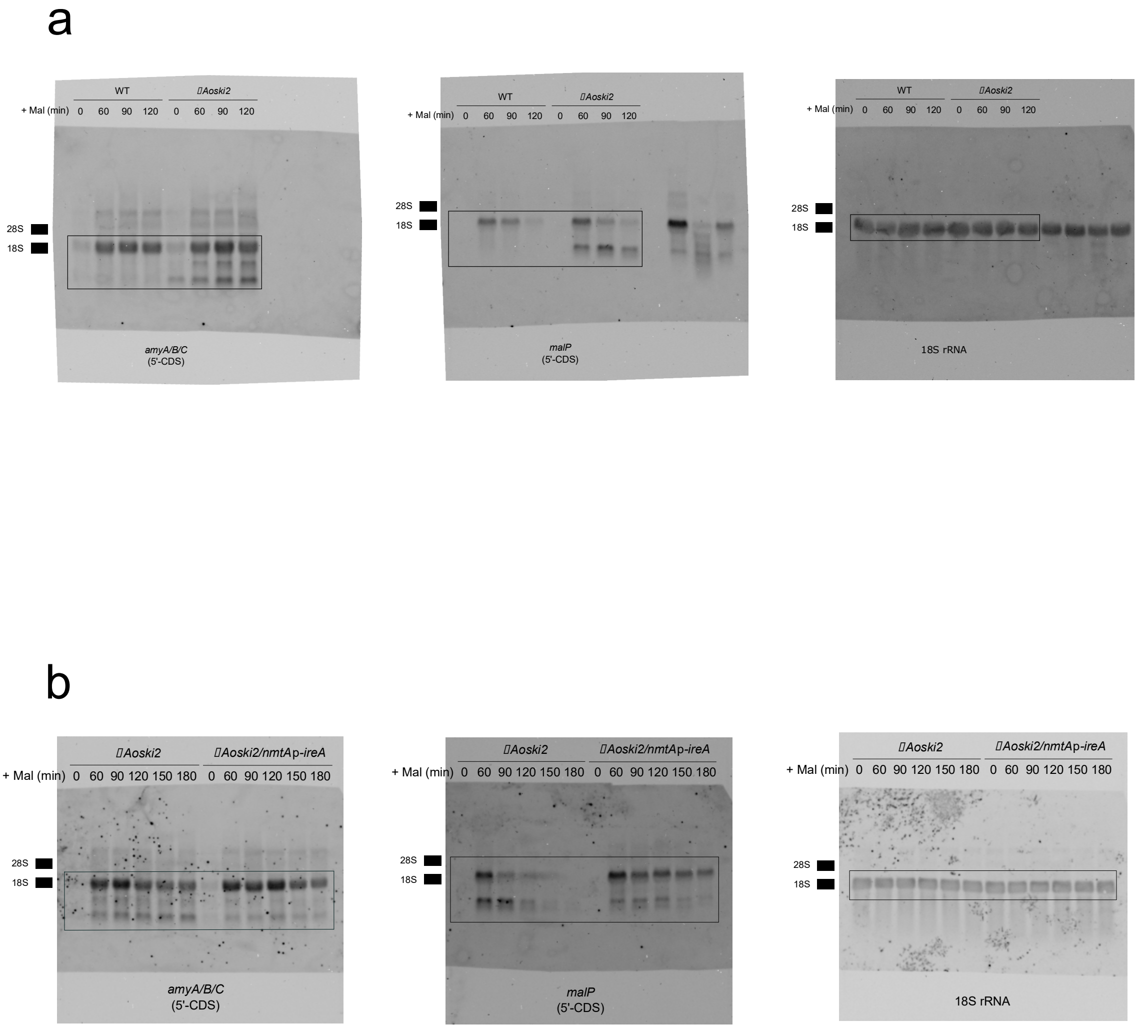

Supplementary Fig. 9. Uncropped and unedited northern blot images for Fig. 4.

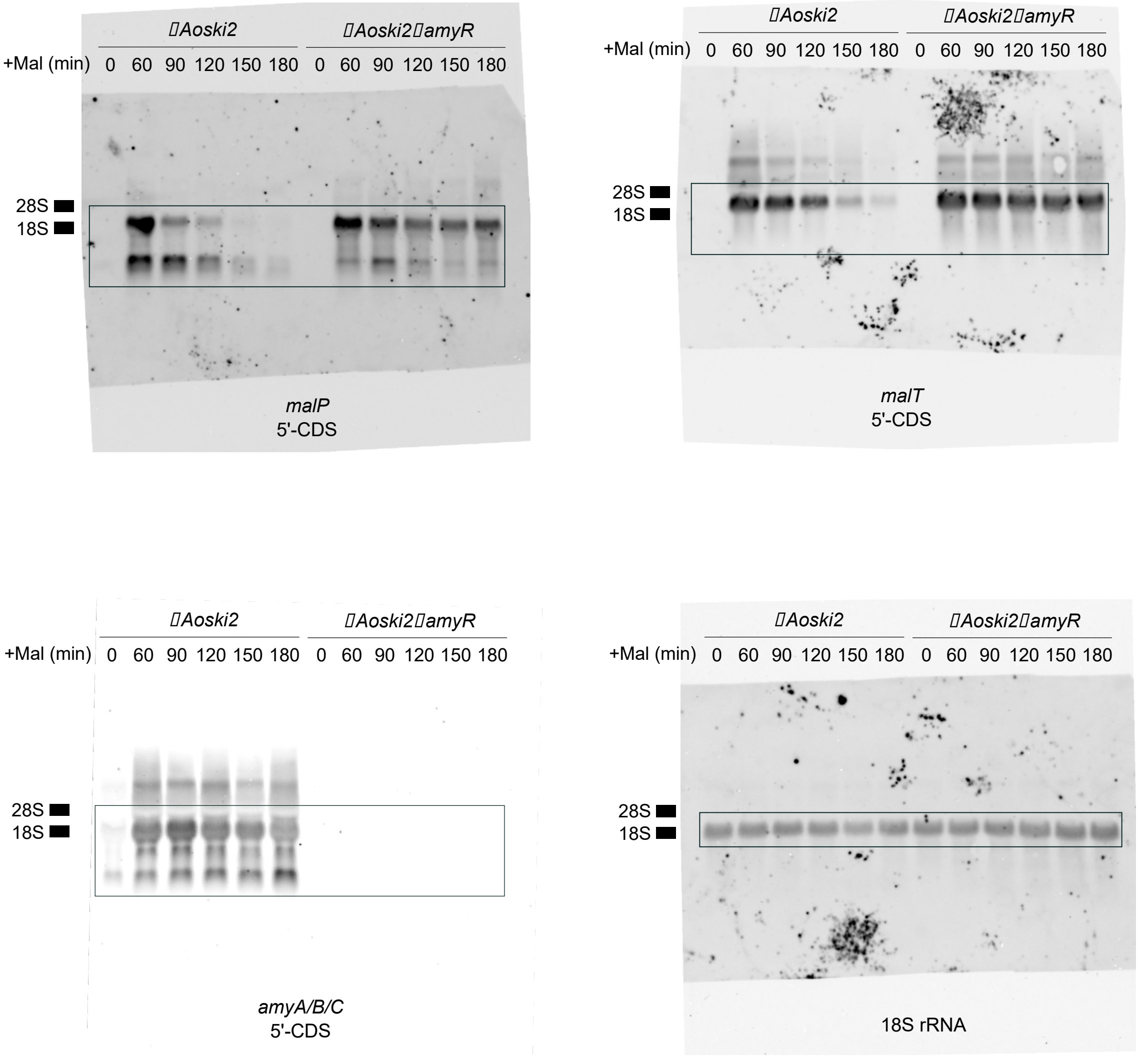

Supplementary Fig. 10. Uncropped and unedited northern blot images for Fig. 5a.

Supplementary Table 1. ***A. oryzae*** **strains used in this study.**

Strain	Strain origin	Genotype	Reference
RIB40			44
NS4	RIB40	*niaD*$^{-}$; *sC*$^{-}$	44
Δ*amyA/B/C*	NS4	*niaD*$^{-}$*;* Δ*ligD::ptrA*; Δ*amyA::sC*; Δ*amyB::loxP*; Δ*amyC::loxP*	21
Δ*amyA/B/C actAp-amyB*	Δ*amyA/B/C*	*niaD::actAp-amyB*; Δ*ligD::ptrA*; Δ*amyA::sC*; Δ*amyB::loxP*; Δ*amyC::loxP*	This study
Δ*amyA/B/C amyB*	Δ*amyA/B/C*	*niaD::amyB;* Δ*ligD::ptrA;* Δ*amyA::sC;* Δ*amyB::loxP;* Δ*amyC::loxP*	This study
Δ*amyA/B/C amyB-FS*	Δ*amyA/B/C*	*niaD::amyFS;* Δ*ligD::ptrA;* Δ*amyA::sC;* Δ*amyB::loxP;* Δ*amyC::loxP*	This study
nmtAp-ireA	NS4	*niaD*$^{-}$; *sC*$^{-}$; *nmtAp-ireA::sC*	19
WT+*hacA*	NS4	*niaD*::*hacA*; *sC*$^{-}$	19
WT+*hacA*^{i}	NS4	*niaD*::*hacA*^{i}; *sC*$^{-}$	19
nmtAp-ireA +hacA	*nmtAp-ireA*	*niaD*::*hacA*; *nmtAp-ireA*::*sC*	19
nmtAp-ireA +hacA^{i}	*nmtAp-ireA*	*niaD*::*hacA*^{i}; *nmtAp-ireA*::*sC*	19
nmtAp-ireA pyrG^{-}	Δ*ligD*::*loxP pyrG*$^{-}$	Δ*ligD*::*loxP*; *niaD*$^{-}$; *nmtAp-ireA*::*sC*; *pyrG*$^{-}$	19
Δ*ligD*::*loxP pyrG*::*niaD*	Δ*ligD*::*loxP pyrG*$^{-}$	Δ*ligD*::*loxP*; *sC*$^{-}$; *pyrG*::*niaD*	48
Δ*Aoski2 nmtAp-ireA*	*nmtAp-ireA pyrG*$^{-}$	Δ*ligD::loxP; niaD*$^{-}$*;* Δ*Aoski2::sC; nmtAp-ireA::pyrG*	This study
Δ*Aoski2*	Δ*Aoski2 pyrG*$^{-}$	Δ*ligD*::*loxP*; Δ*Aoski2*::*sC*; *pyrG*::*niaD*	This study
Δ*Aoski3*	Δ*Aoski3 pyrG*$^{-}$	Δ*ligD*::*loxP*; Δ*Aoski3*::*sC*; *pyrG*::*niaD*	This study
Δ*Aoski2 pyrG*$^{-}$	Δ*ligD*::*loxP pyrG*$^{-}$	Δ*ligD*::*loxP*; *niaD*$^{-}$; Δ*Aoski2*::*sC*; *pyrG*$^{-}$	This study
Δ*Aoski3 pyrG*$^{-}$	Δ*ligD*::*loxP pyrG*$^{-}$	Δ*ligD*::*loxP*; *niaD*$^{-}$; Δ*Aoski3*::*sC*; *pyrG*$^{-}$	This study
Δ*amyR*	Δ*ligD*::*loxP pyrG*$^{-}$	Δ*ligD*::*loxP*; *niaD*$^{-}$; *sC*$^{-;}$ Δ*amyR*::*pyrG*	This study
Δ*Aoski2* Δ*amyR*	Δ*Aoski2 pyrG*$^{-}$	Δ*ligD*::*loxP*; *niaD*$^{-}$; Δ*Aoski2*::*sC*; Δ*amyR*::*pyrG*	This study
Δ*Aoski3* Δ*amyR*	Δ*Aoski3 pyrG*$^{-}$	Δ*ligD*::*loxP*; *niaD*$^{-}$; Δ*Aoski3*::*sC*; Δ*amyR*::*pyrG*	This study

Supplementary Table 2. Primers used for plasmid construction in this study.

Primer name	Nucleotide sequence (5′–3′)
oTAKA381	CGGGGGATCCTCTAGAGGGAAAAAGGATTACGTTGAAGGG
oTAKA382	TGATTACGCCAAGCTTCATGGTGGGGTAACCAAGG
YS3	TTCTGTACGGCCTTCAGGTCGCGGCACCTGCTTTATGGCTGCAACGCCTGCGGAC
YS4	GAAGGCCGTACAGAAATAGAGACCACCACGCGACCCATAAATGCCTTCTGTGGGG
Vector-F	ATGATGGTCGCGTGGTGGTC
Vector-R	ACTAAGCCCTTCAACGTAATCC
PactA-F	ATTACGTTGAAGGGCTTAGTAGCTGGTATTCTTCCCGAACG
PactA-R	GACCACCACGCGACCATCATGATGACTGAATAGGATTACTACG
ski2upsenKpnI	GGAGTAGTAACTAGGTACCGCTGCCTCAGG
ski2upantiBamHI	GCCATCGGGGGACTCTAGAAGTGACTGTCC
ski2downsenPstI	ACCACTCTCTGATCTGCAGTGTCTCACCAA
ski2downantiPstI	TCACATCCCTCTTCTGCAGTTCTTGTGC
ski3upsenBamHI	TGAGCTAGGATCCCAAGTAAGGCAGC
ski3upantiXbaI	CACACCTCCTCTAGATTCGCCTTCTTTTCG
ski3downsenPstI	GATATCTGCAGCTCGTCAGCCCCATTGC
ski3downantiSphI	GTTGCATGCACCGTGTGATTTAGTTCTTC

Supplementary Table 3. Primers used for probe synthesis in this study.

Primer name	Nucleotide sequence (5′–3′)	Application
amyABCfullsen	ATGATGGTCGCGTGGTGGTCTC	*amyA/B/C* CDS probe, *amyA/B/C* 5′-CDS probe
amyABCfullanti	TCACGAGCTACTACAGATCTTGCTACC	*amyA/B/C* CDS probe
amyB5halfanti	AGTGCCGTAGTTTTCGTTCAGAGAG	*amyA/B/C* 5′-CDS probe
glaAfull5sen	ATGGTGTCTTTCTCCTCTTGTCTCC	*glaA* CDS probe
glaAfull3anti	TCACCGCCAAACATCGCTCTGCAC	*glaA* CDS probe
bipAsen	CCAAGCCGTTCACTGCTTGGACTAC	*bipA* CDS probe
bipAanti	ATCAGCCGGAGCAGAGCCATAGAG	*bipA* CDS probe
enoA-NP_Fw	TCCACGCCCGCTCTGTTTAC	*enoA* CDS probe
enoA-NP_Rv	GGTTGACAGAAGTGCGGAAC	*enoA* CDS probe
malP5sen	ATGACTCCAGAGAAGGCCGCTATGTC	*malP* 5′-CDS probe
malP5anti	GCCAGATCCACTGAAGAGCAAAGGGGATAC	*malP* 5′-CDS probe
malT5sen	ATGTCTCCAGCCCCAGTTGG	*malT* 5′-CDS probe
malT5anti	ATACTTCGCAAGCACCTCGTTC	*malT* 5′-CDS probe
18SrRNAsen	CTTGCGCCGGCGATGGTTCATTC	18S rRNA probe
18SrRNAanti	ACCTCTCGGCCAAGGTGATGTACTC	18S rRNA probe

Supplementary method

Synthesis of DIG-labeled probe for detecting the *actA* mRNA using northern blot analysis.

The *actA* cDNA clone identified using the *A. oryzae* expressed sequence tag analysis[1] was used as a template for PCR to synthesize the DIG-labeled DNA probe.

Supplementary Reference

1. Akao, T. *et al*., Analysis of expressed sequence tags from the fungus *Aspergillus oryzae* cultured under different conditions. *DNA Res*. 14: 47–57 (2007).
