# Supplementary material for: Physiological ER stress caused by amylase production induces regulated Ire1-dependent mRNA decay in Aspergillus oryzae
Source: Commun Biol. 2023 Oct 4;6:1009. doi: 10.1038/s42003-023-05386-w (PMC10551036; doi:10.1038/s42003-023-05386-w)
Supplement: Supplementary file 3 — Description of Additional Supplementary Files [file 42003_2023_5386_MOESM3_ESM.pdf]

Description of Additional Supplementary Files

File name: Supplementary Data 1

Description: Source data for boxplot in Supplementary Fig. 2
